# AGE DIFFERENCES IN CLIMATE CHANGE ATTITUDES AND BEHAVIORS

**DOI:** 10.1093/geroni/igad104.2676

**Published:** 2023-12-21

**Authors:** Yeonsoo Shin, Giyeon Kim

**Affiliations:** Chung-Ang University, Seoul, Republic of Korea; Chung-Ang University, Seoul, Republic of Korea

## Abstract

Although previous studies show that older adults are vulnerable to the effects of climate change, the actions of older adults to address climate change have not received much attention. The purpose of this study is to investigate age differences in the perception of climate change and the characteristics of pro-environmental behavior. We analyzed cross-sectional data from a nationally representative sample drawn from the 2022 Korea Social Survey. The sample consisted of 15,252 adults, including younger adults (aged 19-34, n=6,132) and older adults (aged 65+, n=9,120). Pro-environmental behavior included the following actions: using public transportation, recycling, reducing the use of disposable products, and saving water and power. T-test and moderation analysis were conducted. Results showed that the younger group was more likely to be aware of climate change than the older group (F=68.022, p< 0.01). However, older adults were less likely than younger adults to make food waste and save water and electricity more (p< 0.01). There were no age differences in recycling, which is a representative environmental protection behavior. Results from a moderation analysis revealed that younger adults made more effort to recycle in the lower level of climate change awareness whereas older adults made more effort to recycle in the higher level (B = .031, p< 0.01). The findings indicate that older adults may play a crucial role in taking action to respond to climate change and suggest that promoting awareness of climate change among older adults could be an effective strategy to encourage pro-environmental behavior.

